# An unusual case of suprascapular nerve neuropathy: a case report

**DOI:** 10.1186/1752-1947-5-419

**Published:** 2011-08-26

**Authors:** Charalambos P Economides, Loizos Christodoulou, Theodoros Kyriakides, Elpidoforos S Soteriades

**Affiliations:** 1 Cyprus Institute of Biomedical Sciences, 2 Antigonis Street, 2035 Strovolos, Nicosia, Cyprus; 2 Agios Therissos MRI Diagnostic Center, Nicosia, Cyprus; 3 Department of Orthopedics, Aretaieio Hospital, Nicosia, Cyprus; 4 Department of Neurology, Institute of Neurology and Genetics, Nicosia, Cyprus; 5 Department of Environmental Health, Environmental and Occupational Medicine and Epidemiology (EOME), Harvard School of Public Health, Boston, MA, USA

## Abstract

**Introduction:**

Suprascapular nerve neuropathy constitutes an unusual cause of shoulder weakness, with the most common etiology being nerve compression from a ganglion cyst at the suprascapular or spinoglenoid notch. We present a puzzling case of a man with suprascapular nerve neuropathy that may have been associated with an appendectomy. The case was attributed to nerve injury as the most likely cause that may have occurred during improper post-operative patient mobilization.

**Case presentation:**

A 23-year-old Caucasian man presented to an orthopedic surgeon with a history of left shoulder weakness of several weeks' duration. The patient complained of pain and inability to lift minimal weight, such as a glass of water, following an appendectomy. His orthopedic clinical examination revealed obvious atrophy of the supraspinatus and infraspinatus muscles and 2 of 5 muscle strength scores on flexion resistance and external rotation resistance. Magnetic resonance imaging showed diffuse high signal intensity within the supraspinatus and infraspinatus muscles and early signs of minimal fatty infiltration consistent with denervation changes. No compression of the suprascapular nerve in the suprascapular or spinoglenoid notch was noted. Electromyographic studies showed active denervation effects in the supraspinatus muscle and more prominent in the left infraspinatus muscle. The findings were compatible with damage to the suprascapular nerve, especially the part supplying the infraspinatus muscle. On the basis of the patient's history, clinical examination, and imaging studies, the diagnosis was suspected to be associated with a possible traction injury of the suprascapular nerve that could have occurred during the patient's transfer from the operating table following an appendectomy.

**Conclusion:**

Our case report may provide important insight into patient transfer techniques used by hospital personnel, may elucidate the clinical significance of careful movement of patients following general anesthesia, and may have important implications for patient safety techniques, including those outlined in the World Health Organization Surgical Safety Checklist program.

## Introduction

Suprascapular nerve neuropathy is a relatively uncommon cause of shoulder weakness and may be overlooked during physical examinations [1]. The most common etiology of suprascapular nerve neuropathy is nerve compression at the suprascapular or spinoglenoid notch from a ganglion cyst [2]. Other causes include direct nerve trauma, neurinoma, vascular malformations, and, most frequently, injuries related to sports activities [3]. We present a case of a 23-year-old Caucasian man who developed suprascapular nerve neuropathy that may have been associated with the patient's transfer from the operating table following an appendectomy.

### Case presentation

A 23-year-old Caucasian man presented to an orthopedic surgeon with a history of left shoulder weakness of about eight weeks' duration. He complained of pain and inability to lift even minimal weight, such as a glass of water, following an appendectomy. The patient had experienced severe acute pain in the left shoulder and scapula on the third day following his operation; however, he had not paid much attention to it, as the pain gradually subsided within the next two weeks, during which time he was hospitalized for ten days post-operatively for high fever. Following his discharge from the hospital, he noticed left shoulder weakness with an inability to lift even small weights; therefore, he visited an orthopedic surgeon.

**Figure 1:**
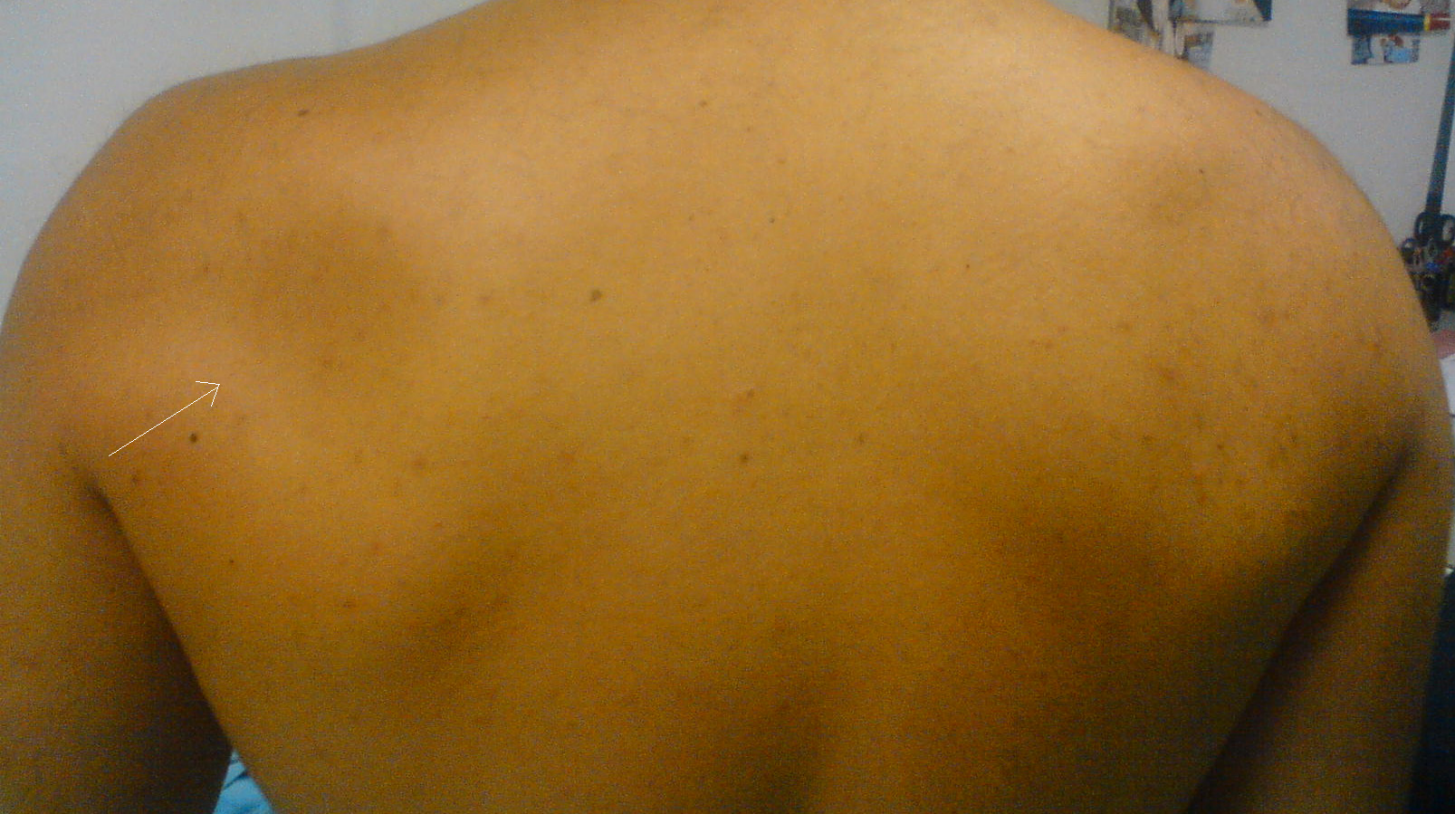
**A picture of both shoulders from the back showing muscle atrophy on the left side (arrow) obtained at the patient's six-month follow-up examination**.

His orthopedic clinical examination revealed that he had obvious atrophy of the supraspinatus and infraspinatus muscles (Figure 1), as well as 2 of 5 muscle strength scores on flexion resistance and external rotation resistance. At his initial presentation to the orthopedic surgeon, an X-ray of the shoulder showed no pathology. On the basis of the clinical examination, the patient was referred for magnetic resonance imaging (MRI) for the detection of possible suprascapular nerve neuropathy secondary to suspected nerve compression by a ganglion cyst.

**Figure 2:**
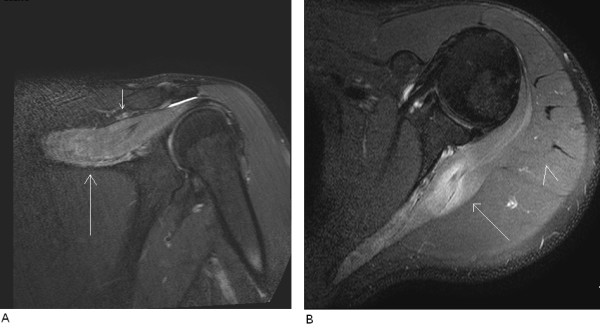
**MRI examination performed three months following the initial symptoms**. (A) Oblique coronal T2-weighted/turbo spin echo fat suppression image of the left shoulder. The long arrow indicates diffuse high signal intensity within the supraspinatus muscle, suggesting denervation changes. The short arrow indicates the suprascapular notch free of pathology. **(B) **Axial T2-weighted/turbo spin echo fat suppression image of the left shoulder. The long arrow indicates diffuse high signal intensity within the infraspinatus muscle, which suggests denervation changes. The arrowhead indicates the adjacent signal from the deltoid muscle, which appears normal.

**Figure 3:**
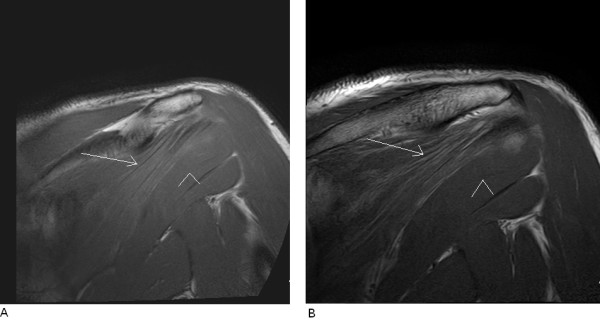
**MRI examination performed at 6 months follow up**. (A) Oblique coronal T2-weighted/turbo spin echo baseline MRI study of the left shoulder. The long arrow indicates diffuse high signal intensity within the infraspinatus muscle, which suggests fatty infiltration due to denervation. The arrowhead indicates the teres minor muscle, which appears normal in contrast to the infraspinatus muscle. **(B) **Oblique coronal T2-weighted/turbo spin echo MRI study of the left shoulder obtained during the six-month follow-up examination. The long arrow indicates diffuse high signal intensity within the infraspinatus muscle. The arrowhead indicates the teres minor muscle, which appears normal in contrast to the infraspinatus muscle. These changes are more extensive, and some loss of muscle volume is also shown, suggesting progression of the denervation effects.

Routine MRI of the shoulder was done using a 3-Tesla scanner three months following the patient's the initial symptoms. A series of T2-weighted/turbo spin echo fat suppression oblique coronal, oblique sagittal, axial T2-weighted/turbo spin echo oblique sagittal, proton density fat suppression, and T2-weighted/gradient echo axial imaging slices through the left shoulder were obtained using a phased array dedicated shoulder coil. These MRI scans showed diffuse high signal intensity within the supraspinatus and infraspinatus muscles on the T2-weighted/turbo spin echo fat suppression images and early signs of minimal fatty infiltration consistent with denervation changes. In addition, no compression of the suprascapular nerve in the suprascapular or spinoglenoid notch and no denervation changes of the teres minor muscle or subscapularis muscle were seen. The MRI findings are presented in Figures 2A and 2B. Following these MRI findings, the patient was referred for electromyography (EMG). EMG showed active denervation effects in the supraspinatus muscle and more prominent denervation effects in the left infraspinatus muscle, which were compatible with damage to the suprascapular nerve, especially the part supplying the infraspinatus muscle. The nerve conduction to the left arm was within normal limits. An MRI examination at the patient's six-month follow-up appointment showed persistent fatty infiltration and loss of muscle volume in both muscles, together with exacerbation within the infraspinatus muscle, supporting our previous findings as well as the EMG results (Figures 3A and 3B).

## Discussion

To our knowledge, this is one of the first reported cases of suprascapular nerve neuropathy most likely attributable to nerve injury that may have occurred during improper post-operative patient mobilization. Although injuries of the nerve are relatively uncommon, the most frequent cause of neuropathy is associated with mass compression usually due to a ganglion cyst or other soft tissue tumors [2]. Other causes may include injuries due to trauma [4] or sports activities [5], repetitive overuse, and iatrogenic causes related to surgical interventions in the nerve area [6-8].

Our case, although it could be categorized as iatrogenic if attributed to improper post-operative patient mobilization, was not associated with surgical interventions in the anatomic region as such cases have previously been reported in the medical literature [6-8]. Instead, it might resemble the mechanisms of injury observed in sports activities [9]. Numerous reports have described suprascapular nerve injury; the pathophysiology associated with stretch or compression that may result in nerve ischemia, edema, micro-environmental changes, and conduction impairment [9].

Our patient was healthy and fit and exercised on a regular basis, and his medical history was unremarkable. On the basis of his recent post-operative pain and weakness, clinical examination, MRI findings, and EMG, we assume that the patient had developed a suprascapular nerve neuropathy. Although the precipitating event was not witnessed, we speculate that the diagnosed nerve injury occurred during his post-operative transfer from the operating table following an appendectomy.

On the basis of this information, we postulate that the mechanism of injury might be attributable to the muscle relaxation associated with general anesthesia that the patient was under during his appendectomy. Such muscle relaxation might have exacerbated the range of shoulder movement (hyperabduction) during his transfer from the operating table, leading to the combination of a traction injury and the so-called "sling effect."

## Conclusion

Based on our assumptions, our presently reported case highlights the importance of using proper patient transfer techniques by hospital personnel, as well as the clinical significance of careful patient mobilization following operations in which the patient has been under general anesthesia, and it may also have important implications regarding patient safety techniques, including the World Health Organization Surgical Safety Checklist program.

## Consent

Written informed consent was obtained from the patient for publication of this manuscript and any accompanying images. A copy of the written consent is available for review by the Editor-in-Chief of this journal.

## Competing interests

The authors declare that they have no competing interests.

## Authors' contributions

CPE compiled the data and obtained and interpreted the MRI studies. TK performed EMG and interpreted the imaging studies. LC performed the clinical examination of the patient. CPE and ESS were the main contributors to the writing of the manuscript. All authors read and approved the final manuscript.
